# Updated roles of cGAS-STING signaling in autoimmune diseases

**DOI:** 10.3389/fimmu.2023.1254915

**Published:** 2023-09-14

**Authors:** Ya Liu, Feifei Pu

**Affiliations:** ^1^ Department of Rheumatology and Immunology, Traditional Chinese and Western Medicine Hospital of Wuhan, Tongji Medical College, Huazhong University of Science and Technology, Wuhan, Hubei, China; ^2^ Hubei Key Laboratory of Skin Infection and Immunity, Wuhan No.1 Hospital, Wuhan, Hubei, China; ^3^ Department of Orthopedics, Traditional Chinese and Western Medicine Hospital of Wuhan, Tongji Medical College, Huazhong University of Science and Technology, Wuhan, Hubei, China

**Keywords:** natural immunity, cGAS-STING signaling, type I interferon, inflammation, autoimmune diseases

## Abstract

Natural immunity, the first line for the body to defense against the invasion of pathogen, serves as the body’s perception of the presence of pathogens depends on nucleic acid recognition mechanisms. The cyclic GMP-AMP synthase-stimulator of the interferon gene (cGAS-STING) signaling pathway is considered an essential pattern recognition and effector pathway in the natural immune system and is mainly responsible for recognizing DNA molecules present in the cytoplasm and activating downstream signaling pathways to generate type I interferons and some other inflammatory factors. STING, a crucial junction protein in the innate immune system, exerts an essential role in host resistance to external pathogen invasion. Also, STING, with the same character of inflammatory molecules, is inseparable from the body’s inflammatory response. In particular, when the expression of STING is upregulated or its related signaling pathways are overactivated, the body may develop serious infectious disorders due to the generation of excessive inflammatory responses, non-infectious diseases, and autoimmune diseases. In recent years, accumulating studies indicated that the abnormal activation of the natural immune cGAS-STING signaling pathway modulated by the nucleic acid receptor cGAS closely associated with the development and occurrence of autoimmune diseases (AID). Thereof, to explore an in-depth role of STING and its related signaling pathways in the diseases associated with inflammation may be helpful to provide new avenues for the treatment of these diseases in the clinic. This article reviews the activation process of the cGAS-STING signaling pathways and its related important roles, and therapeutic drugs in AID, aiming to improve our understanding of AID and achieve better diagnosis and treatment of AID.

## 1 Introduction

Autoimmune disease (AID) causes the immune response in the body to its own antigens owing to body’s dysfunctional immune tolerance, resulting in tissue and organ damage or dysfunction. It usually presents with symptoms such as overactivation of autoreactive B and T cells and the generation of a good deal of autoantibodies (1). Common AIDs includes rheumatoid arthritis (RA), systemic lupus erythematosus (SLE), psoriasis, and psoriasis arthritis. The etiology and pathogenesis of AIDS are unknown, and there is currently a lack of specific treatment methods in clinical practice. However, some non-specific immunosuppressive therapies have drawbacks such as significant side effects, relatively poor therapeutic efficacy, and poor prognosis. Therefore, the key to further improving the prevention and treatment level of autoimmune diseases lies in the study of etiology. Type I interferons (IFN-I) and nucleic acids were considered the crucial effectors in the pathogenesis of AID (2). The cGAS-stimulator of the interferon gene (STING) signaling pathway mediated by cyclic GMP AMP (cGAMP) synthase (cGAS) is a typical natural immune pathway, which can recognize the body’s own DNA and induce the production of IFN-I (3). Therefore, the role of the cGAS-STING signaling pathway in AID has aroused great interest.

In the cGAS-STING signaling pathway, when pathogenic microorganisms infect the human body, the exogenous DNA of the pathogen is recognized by the cytoplasmic nucleic acid receptor cGAS, and a second messenger, cGAMP, is generated using intracellular guanosine triphosphate (GTP) and adenosine triphosphate (ATP). Also, cGAMP can interact with the endoplasmic reticulum localization protein STING, resulting in a change in the conformation of STING, thus recruiting and phosphorylating TBK1 and IKK, and activating NF and interferon regulatory factor 3 (IRF3), respectively. IRF3 enters the cell nucleus, promotes IFN-I expression, and initiates a natural immune response to resist pathogen invasion (4). In addition to recognizing exogenous DNA, abnormal accretion of self-DNA within the cytoplasm could be identified by cGAS, thereby continuously activating the STING signaling pathway and leading to the development of AID (5–8).

Since the finding of cGAS as a cytoplasmic recognition DNA receptor in 2013 (9), numerous small-molecule compounds have been discovered to have therapeutic effects on AID caused by abnormalities in the cGAS-STING signaling pathway. STING inhibitor nitro-fatty acids can inhibit IFN-I production in fibroblasts from patients suffering from stimulator of IFN genes-associated vasculopathy (SAVI) (10). Research has reported that the STING inhibitor SN-011 inhibits the activation of STING mutants related to SAVI disease and can regulate immune disorders in TREX^-/-^ mice, thereby improving the mouse survival rate (11). In TREX^-/-^ mice infected by HSV-1, the small-molecule cyclic peptide Astin C can specifically suppress the inflammatory response caused by the cGAS-STING signaling pathway and cytoplasmic DNA, and significantly reduce the autoimmune inflammatory response in mice (12). Haag et al. (13) reported three highly specific small-molecule inhibitors that target STING proteins: C-176, C-178, and H-151. These small molecules can covalently bind to Cys91 in STING proteins of human and mouse cells, blocking palmitoylation induced by STING activation, hindering the formation of STING polymer complexes, inhibiting downstream immune response activation, and weakening the pathological characteristics of mouse AIDs. This type of compound has strong activity and a low molecular weight, and pharmacological experiments have shown that it can effectively block the signaling pathway of the natural immune response in the body. Hence, they may be highly promising candidate drugs for targeting the cGAS-STING signaling pathway in the treatment of AID. An et al. (14) found that X6, a compound that inhibits the interaction between DNA and cGAS, is more effective than hydroxychloroquine and can significantly reduce the interferon stimulated genes (ISGs) expression in spleen cells of an Aicardi–Goutières syndrome (AGS) mouse model. At the same time, X6 is also significantly better than hydroxychloroquine in reducing the ISGs expression in mononuclear cells in peripheral blood of lupus patients. These results indicate that X6 has a significant advantage in treating AGS and SLE by inhibiting the cGAS-STING signaling pathway activation. Pabosinib, a cyclin-dependent kinase inhibitor, can directly target STING Y167 and inhibit its activation by blocking STING dimerization. Subsequent studies have shown that pabosinib has a protective effect against glucan-induced enteritis and periodic fever syndrome in TREX^-/-^ mice (10). Currently, research has shown that the cGAS-STING signaling pathway is associated with the development and progression of various AIDs (15).

Currently, the treatment methods for AID in clinical practice are still relatively limited, mostly concentrated on the therapy of pathological tissue damage and immune system suppression therapy. Although this type of treatment can have a certain therapeutic effect, it has a greater long-term adverse impact on patients in the long run. In recent years, with the rise in immunotherapy, researchers have attempted to develop targeted therapeutic drugs for different immune signaling pathways involved in AID (16, 17). Given the close connection between AID and the cGAS-STING signaling pathway, inhibitors targeting the cGAS-STING signaling pathway are expected to become candidate drugs for the treatment of AID. This article reviews the cGAS-STING signaling pathway and the functions and applications in AID, with the aim of providing ideas for the research and treatment of AID.

## 2 Properties of cGAS-STING signaling

Natural immunity is a self-defense mechanism in the body through the pattern recognition receptors (PRRs) to recognize the pathogen- and damage-associated molecular patterns and quickly generate immune responses to invading pathogens and related cell apoptosis and tissue necrosis, or damaged tissues; thereby, building the first line of self-defense to fight pathogens in the body (18, 19). PRRs that recognize intracellular nucleotides include nucleotide-binding domain NOD-like receptors, interferon inducible protein 16 (IFI16), retinoic acid-inducible gene-like receptors (RLRs), melanoma deficiency factor 2 (AIM2), and the recently discovered cGAS-STING signaling pathway-related molecules.

cGAS belongs to the nucleotide transferase family and is a sensor of double-stranded DNA (dsDNA) that acts upstream of STING. The unique maleabnormal-21 (Mab-21) domain of cGAS is essential for identifying dsDNA (20). The zinc structure in this domain is embedded in the large groove of DNA, and cGAS binds to negatively charged dsDNA through positively charged amino acid residues and zinc finger structures in DNA. Therefore, the recognition effect of cGAS on DNA is not specific (21), which can accurately identify dsDNA in pathogenic microorganisms. It can also accurately identify the self-DNA that causes abnormalities or leaks in the body’s nucleic acid, including mitochondrial DNA (mtDNA) and nuclear DNA. cGAS usually exists in cells in the form of inactive proteins. When it is combined with dsDNA in a ratio of 2:2, the conformation changes to the active state (4), which further results in the formation of the second messenger 2’, 3’ ring GMP AMP (cyclic GMP AMP, cGAMP) (22, 23) of adenosine triphosphate (ATP) and guanosine triphosphate (GTP) Then the cGAMP is monitored by the dimer cross layer connector protein STING on the layer of endoplasmic reticulum (ER). Fusion with cGAMP activates STING, and then, promotes its translocation of STING protein from the ER to the Golgi apparatus (24). In the whole process of completing this transformation, the STING protein starts to activate and recruit TANK binding kinase 1 (TBK1) from the intermediate of ER and Golgi apparatus. TBK1 can phosphorylate itself and the STING, followed by the interferon regulatory factor 3 (IRF3) and nuclear transcription factor-κB (NF-κB) and signal transduction and activator of transcription (STAT) protein 6 (STAT6) phosphorylation, and cause nuclear translocation of these factors (25, 26). The transfer of phosphorylated IRF3 to the cell nucleus further induces the IFN-I and IFN-stimulating genes expression, initiating an innate immune response (27).

STING is a transmembrane junction protein that is mainly expressed in the endoplasmic reticulum of dendritic cells, macrophages, and T cells and is an important part of the non-specific immunity of abnormal cytoplasmic DNA (28). STING can bind dsDNA directly, and can also be activated by prokaryotic CDNs and 2’5’-cGAMP containing two 3’-5’-phosphodiester bonds upstream of STING, resulting in changes in the conformation of STING (29). After being activated, STING enlists TBK1 to generate STING complex, which can be quickly transported from the ER via the Golgi apparatus located near the nucleosome, and then combined with the nuclear transcription factor IRF3 and induced the latter to phosphorylate and dimerize before entering the nucleus. Moreover, STING could activate IKK to lead the release and translocation of NF-κB into the nucleus. IRF3 and NF-κB in the cell nucleus, two transcription factors B, activate the related genes expression and induce the production of IFN-I and related cytokines. In addition, STING subsequently undergoes phosphorylation, ubiquitination, and other modifications that inhibit its activity and prevent the natural immune response overactivation (30).

**Figure 1:**
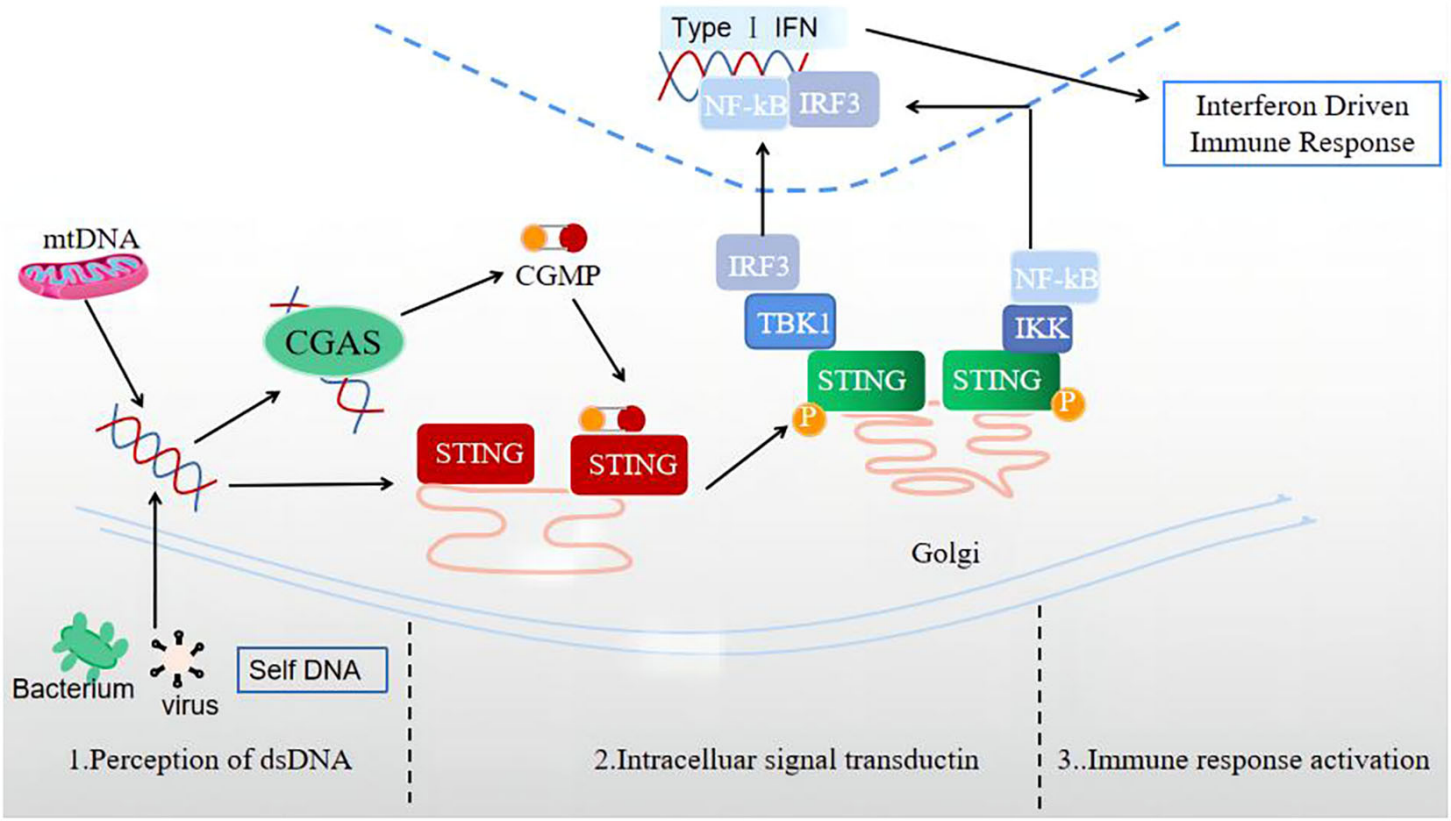
Three stages of signal transmission process of the cGAS-STING signaling pathway: perception of double-stranded DNA (dsDNA), intracellular signal transduction, and immune response activation.

The signal transmission process of the cGAS-STING signaling pathway is normally divided into three stages (**Figure 1**): perception of double-stranded DNA (dsDNA), intracellular signal transduction, and activation of immune response.

1) The perceptual stage of dsDNA. This stage is largely completed by the involvement of the cellular DNA sensor cGAS. Currently, there is no research indicating whether the reported DNA receptors are universal receptors independent of the DNA sequence, except for cGAS. As a broad-spectrum cytoplasmic DNA recognition receptor, cGAS can recognize and adhere to the dsDNA in a DNA sequence-independent manner, catalyzing GTP and ATP to produce 2’3’-cGAMP (31). Notably, during this stage, the cGAS signal can only be activated when the signal triggered by a longer segment of dsDNA exceeds a certain signal threshold (32). In the case of limited cGAS or short dsDNA fragment sequences, cGAS is difficult to activate, providing an important potential protective mechanism for cells.

2) Intracellular signal transduction stage. cGAMP, acting as a second messenger, can activate and bind the STING located in the ER, leading to a STING conformation change (33). Also, STING can bind dsDNA directly, and be activated by prokaryotic cyclic dinucleotides (CDNs) and 2’3’-cGAMP containing two 3’-5’-phosphodiester bonds upstream of STING (29). The combination of STING and cGAMP can promote the transport of STING to the Golgi apparatus. The activated STING can recruit TBK1 to produce the STING-TBK1 complex, then catalyze the phosphorylation and dimerization of IRF3, and TBK1 can also catalyze NF in the nuclear factor to a certain extent- κB’s transposition (33). Activated STING can also directly activate downstream IKK, resulting in NF-κB is released (34).

3) Immune response activation stage. After activation, NF-κB and IRF3 enter the cell nucleus, inducing the expression of IFN-I, ISGs, chemokine, pro-apoptotic genes, and other inflammatory mediators (34). STING, which completes signal transduction, undergoes subsequent modifications such as phosphorylation and ubiquitination, which inhibit its activity and prevent excessive activation of the natural immune response (30).

## 3 Self-DNA and cGAS-STING signaling

The DNA molecules of normal human cells are localized in the cell nucleus or mitochondria, but not in the cytoplasm. Therefore, the existence of DNA in the cytoplasm indicates either invasion of bacterial pathogens, presence of DNA diseases, or damage to the nucleus or mitochondria. As a monitor of DNA sensing, cGAS can indicate threats to the innate immune system. Regarding how the body can avoid inappropriate recognition of endogenous DNA by various PRRs, the possible mechanisms include the following: 1) host receptors may prioritize the detection of pathogen genome structures, including bacterial CpG motifs; 2) The host receptor may be assigned to a limited subcellular region without its own DNA; 3) Many endogenous nucleic acid levels are below the receptor activation threshold (35); 4)The immune monitoring of non-oxidized self-modified DNA is lower than that of oxidized DNA caused by antibacterial physical damage or reactive oxygen species (ROS), thus avoiding the occurrence of autoimmune reactions (36). Similarly, cGAS, as a universal intracellular DNA receptor independent of the dsDNA sequence, also exerts a “double-edged sword” role in innate immunity, reducing the recognition of its own DNA while also recognizing the DNA of exogenous diseases (37). Studies have analyzed the crystal structure of mouse cGAS-DNA complexes and found that long-stranded DNA can bind more cGAS dimers and enhance enzyme activity (38). Subsequently, Chen Zhijian’s research team reported the phase separation phenomenon of cGAS-DNA and found that longer DNA fragments were more effective in promoting droplet formation than shorter DNA fragments, and that the phase transition was sensitive to DNA concentration, confirming that the cGAS activation is dependent on DNA length and concentration (39). Based on this, the Dinshaw team revealed through analysis of human cGAS-DNA complexes crystal structure that there is an additional DNA-binding interface in the catalytic domain of human cGAS that can enhance enzyme activity and liquid-phase condensation (40). These findings partially explain the mechanism by which cGAS prevents fragmented DNA and low-concentration DNA interference when performing intracellular immune monitoring functions. Recently, cGAS is considered a membrane-localized egg binding to plasma membrane lipids via its N-terminal protein. In the resting state, localization of the N-terminal membrane of cGAS can prevent cGAS from translocating to the cytoplasm, avoiding its recognition of trace amounts of DNA within the cytoplasm, thereby reducing activation; therefore, when cGAS is abnormally located, it does not increase its immune response to exogenous diseases (41). Moreover, cell’s own DNA can activate the cGAS-STING signaling pathway through various mechanisms, as described below.

### 3.1 Self-DNA clearance defect

3’-5’-nucleic acid repair exonuclease 1 (TREX1) is an important endogenous DNA exonuclease in cells. It can remove DNA fragments that need to be processed by hydrolyzing damaged DNA, thereby avoiding excessive immune activation and AID. Researchers have found that Trex1^-/-^ mice develop fatal inflammatory diseases around the 8th week after birth. If Irf3 is knocked out at the same time, it can effectively avoid the generation of high IFN-I levels and autoantibodies and prevent the early death of Trex1^-/-^ mice. This result indicates that the autoimmune symptoms of Trex1^-/-^ animals are associated with the generation of IRF3 dependent IFN-I (42). Meanwhile, Trex1^-/-^ mice exhibited an autoimmune inflammatory phenotype associated with increased expression of ISGs. All detectable pathological and molecular phenotypes of Trex1^-/-^ cGAS^-/-^ mice were alleviated, including ISGs induction, the production of autoantibody, abnormal activation of T cell. Even if only one cGAS allele is missing, it can greatly salvage the phenotype of Trex1^-/-^ mice. These results suggested that cGAS inhibition may help prevent and treat certain self-DNA-induced AID (43). Other studies have shown that TREX1 has a lower degradation efficiency for DNA molecules with oxidative damage. In lupus-prone mice, injection of oxidized DNA into the skin can cause injuries like the observation in AID patients (36). TREX1 functional deletion mutations can cause IFN-I dependent AID such as familial chilblain lupus (FCL) and AGS. The manifestations of autoimmune disorders are related to the inability of cells to clear their own DNA fragments normally (44, 45).

Deoxynucleotide ribonuclease II (DNase II) is an acid endonuclease located in lysosomes, nucleus, and cell secretions. The DNase II family is involved in various functions in the human body, such as degradation of the phagocytic dead cells DNA and participation in the growth and differentiation of red blood cells. The function of phagocytes in DNase II^-/-^ mice is damaged, which can cause serious immune deficiency and affect growth and development (46). It is reported that DNase II^-/-^ mice die in the embryonic stage of uterus, and the mouse embryonic liver contains many macrophages with undigested DNA, which indicates that DNase II^-/-^ mice cannot degrade DNA derived from erythroid Precursor cell, leading to the generation of IFN-I, inducing the specific ISGs expression, and leading to embryonic death of DNase II^-/-^ mice (5, 43, 47). However, adult DNase II^-/-^ mice can die from chronic polyarthritis similar to RA [43]. For DNase II^-/-^ cGAS^-/-^ mice, the mice were able to develop normally without obvious arthritis symptoms and showed low levels of anti-dsDNA antibodies. In DNase II^-/-^ cGAS^-/-^ mice, cGAMP expression was not observed, while cGAMP was highly expressed in DNase II^-/-^ Sting^-/-^ mice. Therefore, it can be considered that cGAS is essential for the cGAMP generation in response to the accumulation of their own DNA in the cytoplasm in DNase II^-/-^ cells (43). Through systematic screening of ISGs, studies have identified two siblings and a single male patient with severe membranoproliferative glomerulonephritis, neonatal anemia, and liver fibrosis, all of which are accompanied by an increase in anti-DNA antibodies. A biallelic mutation in DNASE2 was found in these two families, which was related to the loss activity of DNase II endonuclease (48).

Ribonuclease H2 (RNase H2) is a non-nucleic acid sequence-specific DNA repair enzyme to remove unnecessary DNA ribonucleotides. Changes in RNase H2 enzyme function are a common cause of AGS in children and are related to SLE (49). A study using Rnaseh2b^A174T/A174T^ mice as a subclinical disease model found that the transcription of ISGs in mice was upregulated, which is similar to the characteristics of patients with AGS. This inflammatory response depends on the nucleic acid sensor cGAS and its adapter STING and is related to decreased cell RNaseH2 enzyme activity and increased DNA damage (50, 51). Cgas ^-/-^ and Sting^-/-^ mice rescued autoimmune phenotypes and inflammation in Rnaseh2b^A174T/A174T^ mice (50).

### 3.2 Leakage of self-DNA molecules in cells

#### 3.2.1 Mitochondrial DNA stress

Mitochondrial DNA (mtDNA), usually existing in the form of thousands of copies in the cell, is packaged into hundreds of high-level structures called “nucleoids” (48). Mitochondrial transcription factor A (TFAM) binds to abundant mtDNA to regulate the structure, abundance, and separation of “nuclear like” proteins. Studies have shown that a lack of TFAM causes mtDNA stress, and abnormal mtDNA can escape into the cytoplasm, bind to the cGAS, and promote STING-IRF3 dependent signaling to increase the ISGs expression, enhance the IFN-I response, and promote viral resistance (52). Some studies have also shown that the mechanism of cell death determines whether dead cells trigger inflammatory responses or maintain immune silencing and that mitochondria exert a core role in inducing cell death and immune silencing signaling pathways. In a non-alcoholic steatohepatitis mouse model, mtDNA in mouse liver cells can induce Il-6 and TNF-α expression in Kupffer cells (53). Pretreatment with the inhibitors weakened the influence of mtDNA on the inflammatory factors expression. It is worth noting that there are also dysfunctional mitochondria in SLE. A study reported that a decrease in the mtDNA copy number of plasma leukocytes in SLE patients is related to an increase in the disease activity index and an increase in the plasma DNA oxidative damage marker 8-hydroxy deoxyguanosine level (54).

#### 3.2.2 Nuclear DNA

Chromatin has traditionally been regarded as a nuclear entity modulating the silencing and expression of regulatory genes. While, recent studies have shown that, during aging, cytoplasmic chromatin fragments are stripped from the intact primary cells nuclei. This is a terminal cell cycle arrest form related to the proinflammatory response. Some studies have found that cytoplasmic chromatin can activate the natural immune cytoplasmic DNA-sensing cGAS-STING pathway and inhibit the proto-oncogenes activation in acute inflammation. However, it is also related to tissue damage and cancer in chronic inflammation. The cGAS-STING pathway-cytoplasmic chromatin promotes age-related secretory phenotypes. Sting^-/-^ mice have impaired immune monitoring of the oncogene Ras and a weakened tissue inflammatory response induced by ionizing radiation (55). Another study reported that in patients with ataxia telangiectasia, damaged DNA repair promotes the release of nuclear DNA and its accumulation in the cytoplasm, resulting in IFN-I production, and at the same time, leading to the spontaneous formation of extracellular traps in neutrophils (56). In 2017, cGAS was located in the cytoplasm of non-mitotic cells; however, when proliferating cells were in the mitotic stage, cGAS could enter the nucleus and bind to chromosomal DNA (57). Nuclear localization of cGAS may be essential for the antiviral function, as most DNA viruses infect host cells and enter the nucleus, releasing the viral genomic DNA and replicating within the nucleus. Subsequently, cGAS could be transported into the nucleus during the presence of DNA damage in cells and inhibits DNA Homologous recombination repair at the DNA damage site, thereby reducing genome stability and promoting tumorigenesis, which is independent of the function of DNA recognition in the nucleus of cGAS (58). Thereof, further study is required to elucidate the underlying mechanism of cGAS nuclear translocation and its capacity of distinguishing between non-self- and self-DNA in the nucleus. Generally, cancer cells exhibit chromosomal instability. Some of the reasons for cancer metastasis in the human body may be related to chronic DNA leakage from tumor cells in the body were reported (59). DNA leakage activates the cGAS-STING pathway to result in chronic inflammation within cells and helping tumor cells spread to distant organs.

## 4 cGAS-STING signaling in inflammatory diseases

### 4.1 cGAS-STING signaling in infectious inflammation

STING proteins play an essential role in immune defense. Although STING gene-deficient mice survive, they are susceptible to infection by various pathogenic microorganisms. For example, after knocking out the STING gene in mouse embryonic fibroblasts, macrophages, dendritic cells, herpes simplex virus 1 (HSV1), and gram-positive Listeria monocytogenes, bacterial genomes or plasmids cannot effectively induce these cells to produce type I interferons (60, 61). After using *in vitro* short hairpin RNA to interfere with cGAS and STING in human monocyte-macrophage lines, the type I interferons expression level significantly decreased following infection with Mycobacterium tuberculosis in the presence of restricted cGAS or STING expression. This conclusion was also confirmed in mice with cGAS or STING gene deletion mutations; M. tuberculosis could activate STING-related autophagy processes, clearing M. tuberculosis in mice through the lysosome degradation pathway (62). In addition, murine C. trachomatis can activate the STING-related signaling pathway, inducing an anti-C. trachomatis immune response in mice (63, 64). In mice infected with chronic hepatitis B virus (HBV), and the cGAS-STING pathway could inhibit HBV replication in the liver cells of mice infected with chronic HBV. Activating STING protein in mouse liver cells and surrounding immune cells significantly inhibits viral replication in mice, whereas knocking out STING protein in liver-related cells significantly increases the copy number of HBV DNA in mice (65). Moreover, the expression level of STING in chronic hepatitis B patients is lower than that in normal individuals and is correlated negatively with the viral replication level (66). STING not only exerts an essential role in the host’s resistance to dsDNA microbial infections but may also play a significant role in the body’s immune response induced by negative- or positive-stranded RNA viruses. Studies have shown that mice with downregulated STING protein expression in embryonic fibroblasts are extremely sensitive to vesicular stomatitis and murine respiratory virus infections (67). Studies have shown that after HIV (HIV) invades host cells, the body can induce anti-HIV immune effects by activating cGAS-STING-related signaling pathways, thus effectively inhibiting HIV replication in the host. However, when cells are restricted to STING expression, the addition of the synthetic double-stranded RNA (dsRNA) analog polyinosinic: polycytidylic acid does not affect the capbility of the cells to generate type I interferon. In contrast to double-stranded DNA microorganisms, RNA pathogens not only did not significantly induce STING-dependent autophagy, but also did not significantly activate the expression of its associated innate immune genes (24, 68). Therefore, the anti-pathogenic mechanism of STING in RNA virus replication may differ from its mechanism of inhibition of DNA virus replication. In conclusion, the mechanism for STING to regulate RNA viral replication remains further study; however, STING molecules may assume a variety of functions in host cells, including the control of transposon-related tasks, and may affect the post-translational modification of viral proteins. In comparison to wild-type mice, the central nervous system of STING gene-deficient mice can easily cause herpes simplex virus encephalitis when infected with HSV1, and the deficient mice easily develop HSV1 venous infection, but there is no obvious difference in the survival ratio of the deficient and wild-type (WT) mice infected with mucosal infection. During mucosal HSV1 infection, clearance of the virus in mice may not require the involvement of STING, as its impact on the generation of type I interferon is minimal (69, 70). As significant heterogeneity and racial differences exist in human STING genes, further clarification is needed regarding the key role of STING in human pathogenic microorganisms invasion.

### 4.2 cGAS-STING signaling in non-infectious inflammation

The release of endogenous substances from myocardial cells during myocardial infarction will initiate the cGAS-STING signaling pathway to induce the generation of a good deal of inflammatory factors and inflammatory cells, and intensifies the necrosis of myocardial cells. In comparison to WT mice, the early survival rate of myocardial infarction mice lacking cGAS, STING, and IRF3 was significantly improved (71). STING-related signaling pathways also exert a pivotal role in the inflammation of cardiovascular tissues induced by high glucose and high-fat diet in mice. In the above model, weight gain, cardiovascular inflammatory response, reduced glucose tolerance, and insulin resistance of STING-deficient mice improved, suggesting that STING is also involved in metabolic-related inflammatory diseases (72). The STING pathway activation may be an essential regulatory factor in renal injury. In cisplatin-induced acute renal damage in mice, cisplatin can induce the leakage of mtDNA into the cytoplasm through Bcl-2 like protein 4 on the outer layer mitochondrial membrane tubules and activate the cGAS-STING pathway, thus triggering inflammation and exacerbating acute renal failure in mice. However, this process is upregulated in STING gene-deficient mice, and in renal tubular cells *in vitro*, which inhibit the activity of STING protein, can improve the inflammatory response of renal tubular cells caused by cisplatin (73). In mice with acute pancreatitis, the STING-related signaling pathway can sense the DNA of dying acinar cells in the mouse pancreas, further increasing the inflammatory factors generation. Simultaneously, macrophages exacerbate the inflammatory response of the mouse pancreas by overexpressing STING protein (74). A physiological barrier formed by intestinal epithelial cells can separate the intestinal lumen from the internal environment in the host. The STING signaling pathway is associated with the intestinal inflammatory and immune responses of intestinal epithelial cells, leading to fatal sepsis by promoting intestinal epithelial cell apoptosis and destroying the intestinal barrier and the STING protein expression level in mononuclear cells of peripheral blood in septic patients (75). In sepsis mice induced by cecal ligation and perforation, the STING signaling pathway in the intestine is significantly activated, whereas STING gene knockout mice show a reduced intestinal inflammatory response, weakened intestinal permeability, and reduced bacterial translocation. The STING protein was activated in mice by the flavonoid vascular disruptor 5,6-dimethyloxanthenone-4-acetic acid and more significant intestinal cell apoptosis and severe systemic immune inflammatory responses were observed in mice (76).

## 5 cGAS-STING signaling in autoimmune and autoinflammatory diseases

### 5.1 cGAS-STING signaling in SLE

SLE is a prototypic AID with high autoantibodies titers and various organ lesion. Although the exact cause of SLE is currently unclear, accumulating evidence has shown that IFN-I is a primary pathogenic factor in SLE and an important element in the family of cytokines to activate both the innate and adaptive immune systems (77). Moreover, Existing research has shown that SLE is related to nuclease activity deficiency (78, 79). DNase II^-/-^ mice die in the embryonic stage because of the excessive accumulation of IFN-I in their bodies (80), whereas mice with both DNase II and STING deletions can survive without developing lupus (5). These results suggest that lupus caused by DNase deficiency rely on the STING signaling pathway.

The cGAS-STING signaling pathway is associated with the development and occurrence of SLE. Serum dsDNA levels in SLE patients are significantly higher than those in healthy controls (81). Some SLE patients have elevated serum levels of cGAMP, cGAS, and immune-stimulating factors (82), indicating that cGAS may be involved in the process of recognizing and binding to dsDNA to produce cGAMP in patients with SLE, thereby inducing inflammation. However, the subsequent research carried out by Kato et al. shows that the STING agonist 2’3’-cGAMP does not exist in the serum of SLE patients, and dsDNA is stored in apoptosis derived membrane vesicle, which can effectively prevent the degradation of extracellular nuclease and continuously activate the cGAS-STING signal pathway, promoting the release of some inflammatory factors, including IFN and interleukin-6 (IL-6) (81). The STING pathway activation by dsDNA is not necessarily mediated solely by cGAS, there exists a possibility that dsDNA directly activates STING, and the sustained activation of the cGAS-STING signaling pathway is likely involved in cell apoptosis. A study has shown that calcium-related signaling proteins are also associated with regulating the STING pathway in patients with SLE, thereby regulating their immune response (83). The role of cGAS-STING signaling in SLE remains controversial. In a SLE mouse model induced by pristane (TMPD), cGAS and STING defects failed to prevent the mice from getting sick,and led to upregulated generation of autoantibodies and elevated proteinuria levels in mice (84). Overall, there is considerable heterogeneity in the underlying mechanisms of SLE, and the effect of the cGAS-STING signaling pathway in different stages of SLE pathogenesis varies largely depending on the type of SLE animal model being studied and the stage of disease development.

Administration of the calcium ion inhibitor, dipyridamole, the levels of inflammatory factors mediated by T cells in patients with SLE significantly decreased. SLE animal model experiments also supported this result, suggesting a common molecular pathway between calcium ions and IFN in patients with SLE (85). Using calcium ion chelating agents, Zhang et al. found that inhibition of the calcium signal transduction using calcium ion chelating agents was able to suppress macrophage activation induced by the DNA-dependent activator of interferon regulatory factors (DAI) in SLE (86). These results suggest that calcium-related signals are associated with the STING pathway and modulate the immune responses in patients with SLE.

SLE pathogenesis is associated with enhanced generation of IFNα from lupus monocytes induced by augmented activation of STING pathway, and enhanced expression of STING and subsequent production of IFNα by monocytes were downregulated by the suppression of the mTOR pathway (87). We propose that the cGAS-STING pathway is versatile in multiple organs and can promote the overall SLE progression through impertinent sensing of cytosolic self-DNA (8). These suggest that IFIT3 may contribute to the overactivation of cGAS-STING signaling pathway in monocytes of human SLE, laying the foundation for IFIT3 to be a new therapeutic candidate for blocking type I IFN and other proinflammatory cytokines generation via the cGAS-STING signaling pathway in SLE patients (88). Further investigation will disclose the importance of these molecules in SLE development and progression.

### 5.2 cGAS-STING signaling in RA

RA, a chronic AID character by destructive and symmetrical joint lesions and synovitis (89). The expression of dsDNA in the cytoplasm of fibroblast-like synovial cells in RA patients increases and that the dsDNA expression and cGAS is related to the severity of rheumatoid synovitis. After knocking out cGAS or STING in RA patient cells, the expression of cytokines decreases (90), indicating that the cGAS-STING signaling pathway regulates the inflammatory response in RA. Another study found that cGAS promoted the inflammatory response of RA fibroblast-like synovial cells by activating extracellular regulated protein kinase (ERK) and protein kinase B (Akt) signaling pathways (91). Although the AKT signaling pathway activation may inhibit downstream activation of the STING pathway (92), whether cGAS can activate of the ERK and AKT signaling pathways in RA is related to the regulation of activation of cGAS-STING signaling pathway. In summary, the cGAS-STING signaling pathway is associate with the occurrence and development of RA. Further study will investigate RA pathogenesis and clinical treatment options.

### 5.3 cGAS-STING signaling in Aicardi–Goutières syndrome

Aicardi-Goutières syndrome (AGS) is an autoinflammatory disorder connected with spontaneous IFN production without virus infection (93). Studies have shown that the pathological mechanism of AGS is tightly associated with the cGAS-STING signaling pathway (94). AGS is caused by the mutation and deletion of some nuclease genes, which leads to the reduction or loss of nuclease activity, and a good deal of nucleic acids accumulate STING-TBK1-IRF3/NF-κB in the cytoplasm, the excessive activation of these signaling pathways ultimately results in a significant increase in IFN-I levels (43). It has been confirmed that patients with pulmonary and vascular syndrome have mutations in exons of STING (34) and that primary biliary liver disease is also related to the cGAS-STING signaling pathway activation (95).

### 5.4 cGAS-STING signaling in vasculopathy with onset in infancy

SAVI is a chronic inflammation and vascular disease caused by increased IFN-I signaling because of mutations in genes associated with STING activation. Reports of SAVI cases, both domestically and internationally, are generally related to mutations in the STING coding gene (TMEM173), with clinical manifestations including neonatal inflammatory pulmonary disease, skin vascular disease, and decreased activity tolerance (96). In related SAVI mouse model, the excessive cGAS-STING signaling pathway activation can result in immune system dysfunction, aseptic inflammation, and severe pneumonia in mice, similar to the symptoms observed in patients with SAVI (97). Mutations in the STING coding gene result in the overactivation of STING, inducing a large amount of IFN-I production. After binding to interferon receptors, IFN-I is further cascaded and activated through the Janus kinase (JAK)-STAT (JAK-STAT) signaling pathway, leading to inflammatory storms and causing severe and persistent damage to STING overexpressing tissues (98, 99).

### 5.5 cGAS-STING signaling in psoriasis

Psoriasis, a chronic inflammatory skin disorder, is usually accompanied by complications, including RA, autoimmune Thyroiditis and other AIDs and is related to adaptive and congenital immune responses (100). DNA oxidative insult in the skin of psoriasis patients worsens, and levels of circulating free DNA (cfDNA)in the plasma of psoriasis like mice increase. Additionally, STING and its associated genes are increased in the skin of patients (101–103). Preliminary mechanism research shows that STING, as an autoDNA sensor, induces inflammatory response in macrophages and keratinocyte of psoriatic skin and mediates TNF-α or H_2_O_2_ release by immune cells, generated TNF-α, H_2_O_2_ can promote the discharge of DNA into the cytoplasm of keratinocytes and inhibit the degradation of STING protein induced by dsDNA (102). In a study on the *in vivo* and *in vitro* anti-psoriasis effects of H-151, a STING antagonist, it was found that local administration of H-151 can alleviate imiquimod induced skin damage in psoriasis and alleviate inflammation by inhibiting the STING/NF-κB in keratinocyte and signal transduction of immune cells, suppresses skin inflammation, and alleviates the symptoms of psoriasis (103).

### 5.6 cGAS-STING signaling in non-alcoholic steatohepatitis

The current view is that non-alcoholic steatohepatitis (NASH) is an autoimmune disease caused by abnormal mitochondrial DNA released by liver Kupffer cells against necrotic liver cells (104). Cell necrosis releases mitochondrial DNA, which leads to the generation of TNF-α and proinflammatory factors, through the cGAS-STING signaling pathway, is a committed step for the disease to produce persistent chronic inflammation. WT mice treated with STING activators were more sensitive to steatohepatitis than STING-/- mice, indicating that the cGAS-STING signaling pathway exerts an essential role in the NASH induction. However, it is a long way to go for us to better understand the NASH pathophysiology and underlying molecular mechanisms.

### 5.7 cGAS-STING signaling in inflammatory bowel disease

IBD includes ulcerative colitis and Crohn’s disease, and its pathogenesis is not totally clear. Currently, the mainstream view is that many factors such as genetics, environment, gut microbiota imbalance, and host mucosal immune dysfunction jointly promote the development and occurrence of IBD (105). The special functions of STING in the human gut remain unclear, but STING-deficient mice are prone to experimental colitis (106). Another study suggested that the incidence of spontaneous colitis in STING-deficient mice was obviously shorter than that in the control group (107). Ahn et al. (107) and Balci et al. (108) observed that IL-10 deficient mice showed severe spontaneous colitis and intestinal polyps within 10 weeks, whereas IL-10 and STING dual-gene-deficient mice didn’t show a similar phenotype after 19 weeks. Further research found that only 10% of IL-10 and cGAS dual-gene knockout mice formed intestinal polyps, indicating that the cGAS-STING signaling pathway exerts an indispensable role in IL-10 related colitis in mice.

In a colitis model induced by sodium dextran sulfate (DSS) in mice, compared with WT mice, the colitis symptoms of STING mutant and cGAS^-/-^ mice were obviously reduced, and that the expression level of STING protein is increased in colon M1 macrophages of both DSS-treated WT mice and IBD patients. STING agonists are capable of promoting the transformation of M2 macrophages to M1 macrophages, suggesting that the cGAS-STING signaling pathway may regulate intestinal immunity by regulating colon macrophages (109).

## 6 cGAS-STING pathway small molecule inhibitors

Since the cGAS-STING signaling is involved in the innate and adaptive immunity in human body, search for and identification of specific small molecules to suppress or reduce cGAS-STING signaling activity could be used as a novel approach in control of various human diseases, like AIDs, NASH, or even cancers. For example, Vincent’s team screened cGAS small-molecule inhibitors using a high-throughput sequencing technology and revealed that RU.521 can effectively inhibit cGAS activity in Trex1 deficient AGS mouse bone marrow macrophages and RAW264.7 macrophages, significantly inhibiting the half inhibition rate of type I IFN expression (110). The STING pathway activation requires palmitoylation of cysteine 88/91 (Cys88/91) in the STING protein N-terminal transmembrane region using mass spectrometry analysis and alanine scanning (111). Based on this, Haag et al. further screened and identified 2 derivatives of nitrofuran (C-176, C-178) using cytochemistry. Trex1 deficient mouse experiments revealed that both C-176 and C-178 can reduce the downstream effector molecules (such as IFN-β) of the STING pathway (13). In addition, the natural compound AstinC extracted from the medicinal plant astrogate tubers from plant cyclic peptide compounds were screened. AstinC competitively binds to STING protein with 2’3’-cGAMP and inhibits STING recruitment of IRF3, which can significantly alleviate multiple organ damage caused by excessive type I IFN in Trex1 deficient mice (12). We expect to discover new regulatory molecules and mechanisms underlying the STING pathway in future.

At present, there are no effective drugs for rare AIDs such as AGS and SAVI. Targeting the cGAS-STING signaling pathway alleviates body damage caused by type I IFN and regulates tissue inflammatory damage caused by IL-6 and TNF. The research on the developing cGAS-STING pathway small molecule inhibitors by pharmaceutical companies such as Celgene is in the pre-clinical research stage, and it is believed that they will soon enter the clinical trial stage, which will also benefit patients with AIDs (112).

## 7 Perspectives

Since the first identification of cGAS in 2013, the cGAS-STING signaling pathway has become a popular research topic both domestically and internationally. Recently, multiple studies have delved into key molecules of the pathway, gradually unraveling how host cells induce IFN-I to resist invasion by sensing pathogen nucleic acid when the body encounters bacteria or diseases. This review briefly elaborates on the mechanism of the cGAS-STING signaling pathway triggered by self-DNA and potential drugs and molecules targeting self-inflammation and AID in this pathway. In fact, cGAS and STING inhibitors have broad therapeutic potential and can be used as alternatives to supplement existing treatment strategies or even as standalone treatment options. CGAS and STING inhibitors not only have therapeutic value for autoimmune inflammation and AID, such as AGS, FCL, SAVI, and SLE, but also have potential therapeutic value for more common diseases induced by abnormal cGAS-STING pathway activation, such as non-alcoholic steatohepatitis, chronic obstructive pulmonary disease, and Parkinson’s disease. The aforementioned targeted drugs mostly remain in the animal experimental stage and have shown good therapeutic effects in animal models; however, further experiments may be hindered by non-target effects, such as increasing the risk of a sense of opportunity. It is worth noting that, theoretically, compared to existing therapeutic drugs that block the downstream signaling pathways of IFN-I (such as JAK inhibitors), cGAS and STING inhibitors may have more advantages, as other signaling pathways that recognize nucleotides in the cytoplasm are relatively complete. In addition, negative mediation of the cGAS-STING pathway can also reduce the expression of other inflammatory factors, including TNF-α and IL-6, reduce cytokine storm and tissue damage. More and more details about the cGAS-STING pathway molecular mechanisms are being elucidated gradually; however, there are many questions remain. In most studies, cell lines and mouse models were used; however, there are significant differences between the two, and they pose significant challenges in human clinical trials. Therefore, a further in-depth understanding of the molecular signaling mechanisms activated by STING will provide an essential potential direction for the treatment of infections, cancer, and AID.

## 8 Conclusion

The cGAS-STING signaling pathway, can lead inflammatory storms associated with immune escape,mitosis and autophagy The abnormal activation of cGAS-STING signaling pathway modulated by the nucleic acid receptor cGAS is tightly associated with the development and occurrence of autoimmune diseases (AID). Furthermore, CGAS and STING inhibitors also have therapeutic value for autoimmune inflammation and AID, such as AGS, FCL, SAVI, and SLE. To better prove this viewpoint, further studies are no doubt needed to be performed to study the effect of the downstream of cGAS, such as IFIT3, IL-6, TNF-a, IL-10, in AIDs. And, the therapeutic drugs’ efficacy targeting the cGAS-STING pathway from the following aspects, such as STING agonists, cGAS inhibitors, STING antagonists, Autophagy modulators, etc., should also be reviewed and explored. Therefore, a further in-depth understanding of the molecular signaling mechanisms activated by STING will advance the discover of new treatment candidates for infections and AID.

## Author contributions

YL: Writing – original draft, Writing – review & editing. FP: Writing – original draft.
